# Missed Nuances in Tuberculosis Research: Reflections from a Tibetan Scholar

**DOI:** 10.1177/27551938251314654

**Published:** 2025-02-06

**Authors:** Nawang Yanga

**Affiliations:** 1 Graduate Program in Health Policy and Equity, York University, Toronto, Canada; 2 Dahdaleh Institute for Global Health Research, York University, Toronto, Ontario, Canada

**Keywords:** decolonization, global health, tuberculosis, Global South, refugees

## Abstract

Acknowledging and valuing the lived experiences of scholars from the Global South is crucial for more nuanced, refined, and equitable approaches to and interpretations of research. The sheer lack of Tibetan scholars authoring and leading studies within the Tibetan diaspora is especially concerning. The paucity of Tibetan scholars in tuberculosis (TB) discourse is a marker and product of the colonization of academic global health and of global inequities in opportunity and credibility. The lack of Tibetan voices, advocates and scholars alike, in the TB discourse creates ambiguities and misinterpretations, and a general unwillingness to dig deeper into the social, cultural, economic, and historical contexts under which TB thrives in this community. It also symbolizes the lack of opportunity faced by many scholars based in the Global South. Efforts to decolonize global health must also parallel efforts to address other related injustices.

## My Introduction to Tuberculosis

I was born in Nepal in a family of displaced Tibetan refugees. After the invasion of Tibet, my grandparents escaped Tibet on foot, into bordering India and Nepal, where my parents were born. In my six years of living in Nepal and seventeen years in Canada, I recall several immediate family members, other relatives, and general members of the Tibetan refugee community speaking about their experiences with tuberculosis (TB). In blissful ignorance, I assumed that TB affected all communities in the same way. I assumed that everyone had a family member or relative who had TB. In fact, my own mother had TB during her time in Nepal. She recently told me a story of her journey to find the “best TB medication” possible in Nepal. She mentioned that her financial stability and familial support were two major factors that got her through such difficult times. With furrowed eyebrows she said (in Tibetan), “Oh yeah, so many people in the Tibetan community had TB. In fact, when I was on the taxi ride to get to the TB hospital, there were four to five other Tibetans I met along the way going to the same place.” My brother-in-law added that many people in his Tibetan settlement in Pokhara, Nepal had gotten TB. The nonchalance with which my mother and brother-in-law spoke about their lived experiences with TB was alarming and, truthfully, saddening. It is an unfair reality that plagues millions of people every year. Unfortunately, it is also often a neglected one.

## Tuberculosis in Tibetan Refugee Settlements in India

After the annexation of Tibet in the late 1950s, approximately 80,000 Tibetans fled into neighboring India, with permission from then Prime Minister, Jawaharlal Nehru, following the footsteps of their leader, the 14th Dalai Lama.^1^ The Tibetan peoples were afforded small plots of land during this time, and currently there are about 45 Tibetan settlements across India, Nepal, and Bhutan.^2^ Although TB is a one of the leading public health challenges for the Tibetan diaspora in India, data from this community are sparse and lacking. Based on the little data that exists, the TB situation in the Tibetan refugee community in India is dire. A 2016 study found an incidence of 431 cases per 100,000 persons among Tibetan refugees in India.^3^ During that same year, the incidence for the general Indian population was 181 cases per 100,000 persons. A 2019 article found a prevalence rate of 853 per 100,000 in schoolchildren across 11 Tibetan schools in India.^4^

The Tibetan government-in-exile, also known as the Central Tibetan Administration (CTA), is not recognized by any state and thus exists as a de facto government in India. While the Government of India (GoI) has given the CTA a considerable amount of autonomy, the GoI has the ultimate authority. The CTA Department of Health's TB Control Program has seen a considerable amount of success. However, like other infectious disease programs from low- to middle-income countries (LMICs), it is reliant on collaborations with institutions in the Global North for funding and resources. The Zero TB in Kids (ZTB) initiative, for example, is a project aimed at addressing TB in Tibetan refugee children in India. It is a collaborative project between Johns Hopkins University based in the United States and the CTA, based in India.^5^

For a few years now, I have been working toward studying the TB crisis in Tibetan refugee settlements in India from a critical, social science perspective. Despite alarming numbers of TB cases in Tibetan refugee settlements in India, I have noticed the clear lack of literature on this very topic. There is simply not enough literature related to the TB situation in Tibetan communities in India and, in particular, related to its social determinants.

The existing literature is situated in biomedical and/or quantitative (positivist) research, often neglecting the social determinants of TB. Even more concerning is the scarcity of health literature *authored and led by* Tibetan scholars themselves. The paradoxical nature of researchers from the Global North leading projects on peoples situated in the Global South contributes immensely to the marginalization of voices from the Global South. In the case of TB in Tibetan refugee settlements in India, the result of this problem is clear. We fail to humanize persons with TB and their social, cultural, financial, and historical contexts.

## Tuberculosis: A Global Health Inequity

The vast majority of people with TB live in LMICs. In fact, 80 percent of people with TB and TB-related deaths occur in LMICs, with India making up 25 percent of the global TB burden.^6^ Even with high-income countries (HICs) with low TB burden, the burden of TB falls on pocketed communities including but not limited to refugees, migrants, prison populations, and the homeless. A recent bibliometric analysis of TB research between 2007–2016 found that, despite their very low TB burden, the United States accounted for 18.4 percent of all publications, with India making up 9.7 percent of all publications.^7^ Despite TB's disproportionate burden on Global South, the Global North continues to predominate in knowledge production and dissemination. Furthermore, collaborations between countries occurred most frequently between the United States and LMICs, while collaborations between LMICs were much less frequent. This can be explained by the lack of funding for TB research and development in LMICs, who then rely on HICs for research funding.

The practice of scholars from the Global North—places often less burdened by TB—who travel to the Global South to collect data in communities that are heavily burdened by TB, and then return to their home countries to analyze the data without meaningful collaboration or input from the host communities, is a predatory practice, called “helicopter research” or “parachute research.” This practice mirrors colonial practices of exploitation of local knowledge with little acknowledgement of local communities. To engage in decolonial practices, researchers must be intentional in their research design. This includes mutually beneficial research designs, respecting local rules and norms, continuous and long-term involvement from and with local communities, and dissemination of research locally, among other considerations.^8^

## How Exactly do we Decolonize Global Health?

Efforts to decolonize global health are becoming increasingly popular. Decolonization is a process of undoing the various social and cultural impacts of colonial power that are embedded in all parts of life, from our institutions to the languages we speak. It equally involves emphasizing and making known the value of local ontologies and experiences. This includes the decolonization of global health research, policy, programing, and so much more. But over the years, decolonization has been framed and defined by the dominant Global North, leading to its depoliticization and reduction to a mere buzzword or a checkmark.^9^ For example, a survey of pre- post-doctoral candidates in a multi-university program in global health research found that trainees from LMICs were less aware of the concept of decolonizing global health than trainees from HICs.^10^ This discrepancy is problematic because it shifts the focus from core issues, and risks becoming a tokenistic process. Instead of driving change, it becomes passive, failing to address systemic inequities it seeks to rectify. Kwete and colleagues^11^ argue that remnants of colonial “legacies” in global health is a major barrier for the process of decolonization. Adhikari and colleagues^12^ envision equal access to opportunities in recognition, research and publishing, training, salaries, and access to international travel regardless of country of residence as the way forward in decolonizing global health. McCoy and colleagues^13^ write that the decolonization of global health is a process of challenging and destroying power imbalances and how they are created and sustained. Forsberg and Sundewall^14^ argue that calls for decolonization should be understood as calls for “. . . equitable partnerships and a better power balance between the ‘South’ and the ‘North’”.

Decolonization is a complex but ongoing process that extends beyond academic discourse and rhetoric. While writing and debating about decolonization is important for many reasons, decolonization involves doing. It requires tangible, operational, and systemic changes in current practices. The “buzzwordification” of decolonization in global health adds to the lack of progress in addressing underlying power dynamics and inequities.^8^ Although the foundational elements of decolonization, particularly in global health, travel across space and time, they are also context dependent. In the Canadian context, for example, decolonization involves acknowledging and rectifying the cruel injustices faced by Indigenous peoples at the hands of European settlers. The Truth and Reconciliation Commission (TRC) highlighted the need for reconciliation through informing Canadians about the horrors of the residential school system and its ongoing downstream impacts on the well-being of Indigenous peoples.^15^ The TRC includes 94 Calls to Action that range from education to health. Decolonizing health in Canada can include projects incorporating traditional Indigenous healing practices into Western medicine in clinical settings, for example. In the context of Global Health, then, decolonization poses different meanings, focusing on issues that transcend borders and emphasize international collaboration. It both involves the undoing of colonial legacies and the uplifting of local and Indigenous ontologies.

The issue of tuberculosis in Tibetan refugee communities in India is critical for several reasons: (*a*) TB is a pressing public health issue in the Tibetan diaspora in India, (*b*) there is insufficient literature or research globally on the topic despite the urgency of the issue, (*c*) most scholarship that does exist is often lead or authored by non-Tibetan authors with little involvement from the Tibetan community, and (*d*) the existing scholarship authored by the members of the Tibetan diaspora is funded by external organizations from the Global North. I believe that each of the four components is indicative of the colonial nature of global health and that, in order to eradicate TB in this community, the members of the community must be involved in programing and research efforts, and more scholars and leaders who are themselves Tibetan are needed to join the work of solving these issues.

## Authorship and Funding as Colonial Markers

The dominance of scholars from the Global North in the field of global health research exemplifies enduring colonial legacies. A cross-sectional analysis of 197 publications related to the decolonization of global health revealed that 75.1 percent of these articles had authors affiliated with HICs, while only 4.2 percent featured authors from LMICs.^16^ Additionally, the most prevalent category consisted of publications authored exclusively by individuals from HICs, accounting for 70 percent of all articles, followed by those with a combination of authors from HICs and LMICs, which constituted 22.3 percent of all articles. The underrepresentation of scholars form the Global South include limited research infrastructure due to inadequate funding, the dependence on HICs for funding, and the geographical and social proximity that Global North scholars have with high-impact global health journals, many of which are based in HICs.^17^

A shortage of publications by authors from LMICs in what the Global North deems reputable journals, a lack of academics working within their local communities, and insufficient first authors from HICs in HIC–LMIC collaborative projects all highlight broader issues of inequity in Global Health. When scholars from the Global South struggle to publish in “reputable” and “high-impact” journals, they are also stripped of future opportunities for funding, promotions, employment, and awards. When scholars and institutions from the Global South depend on Global North actors for funding, we perpetuate colonial power asymmetries. This vicious cycle further marginalizes these scholars and benefits scholars from the Global North. Disparities in access to opportunities are a key obstacle to equitable global health research.^10^ Opportunity encompasses opportunity for funding, workshops, training, knowledge, and much more.

As Abimbola details, researchers in HICs are often the source of the funds and research agenda, while LMICs are where the actual research is conducted.^18^ Inequitable opportunity to gain funding is yet another marker of global health inequity for academics. For example, out of 164 studies funded by the National Cancer Institute (NCI) of America between October 2015 and September 2019, 97 percent of the grants were held by U.S. institutions. Out of almost 2,500 publications resulting from the 164 grants, 51 percent did not include authors affiliated with a LMIC. Disparities in funding can also be complicated by political instability and precarity, as observed in 2013 where the nomination for the Kochon Prize theme of “[TB] in conflict and refugee areas” of the Tibetan TB Control Programme of the CTA based in Delek Hospital in India was disapproved by the former World Health Organization (WHO) Director General Margaret Chan.^19^ The WHO disapproved the nomination because they could not award a “. . . prize to an institution that is dependent on an authority that is not recognized by the United Nations”.^20^ Despite almost 60 successful years after its inception, the CTA experiences major barriers in funding from external organizations (particularly from the Global North) that stem from its precarious nature. Its inability to adhere to the standards of the Global North hindered their opportunity to be in the running to receive funding from the WHO, despite the clear need for it and the urgency of the TB crisis in this pocketed community.

## Missed Nuances and Chances for a More Thorough Understanding

I see a pressing need for more Tibetan scholars to engage in research about Tibetan people and issues. This is crucial for cultural sensitivity, preserving authenticity, and agency to represent and interpret their own history and culture. Tibetan scholars naturally have a deeper understanding of their cultural practices, history, and language, which may lead to a more nuanced perspective that may be missed by non-Tibetan scholars. I am not arguing against collaboration between Tibetan and non-Tibetan scholars or the exclusion of non-Tibetan authors from conducting work; instead I wish to encourage projects conducted on Tibetan populations in collaboration with Tibetan peoples throughout the entirety of the project, and most importantly with Tibetan academics. A lack of Tibetan scholars engaging in Tibetan topics may lead to inaccuracies and reliable and meaningful details and perspectives. I suggest a participatory action research (PAR) framework as an alternative to such tensions. As developed and articulated by Fals-Borda and Freire, PAR can be utilized as a decolonial methodological tool in Global Health.^21,22^ The PAR methodology is rooted in social justice, both as a principle and an aim. It combines “academic–expert” voices with local and lived experiences. Its fundamental principle is the integration of local ontologies and expertise into the research project from design to dissemination. It values the research subject and positions them as “experts,” rather than mere subjects or participants. It involves constant collaboration to shape and guide the research agenda, rather than the researcher imposing their research agenda. PAR parallels decolonial methods in many ways, particularly in its desire to dismantle systems and structures that are inherently beneficial for the person holding the power and to involve local knowledge holders. Fals-Borda emphasized the multifaceted role of a researcher, not only as one who seeks knowledge but as one who must support and empower the community they study.^23^

PAR has been utilized in TB-related programs and research. A photovoice-based PAR project explored the lived experiences of women with drug-resistant TB in Mumbai, India.^24^ In this particular study, participants were given the freedom to “cocreate” their own experiences through photographs. Throughout the entire research process, participants were involved. This included participants identifying the duration and time of focus group interviews and active engagement during the researcher's interpretation and analysis of data. Another PAR-based program was created for and in collaboration with a large Aboriginal family (14 peoples) in a rural town in Australia dealing with multiple, repeated episodes of TB. The purpose of this project was to create a sustainable program to improve access to nutritious food and housing hardware, both of which are social determinants of TB.^25^ How might utilizing a PAR methodology improve the handful of TB-related studies on the Tibetan communities?

Overcrowding in monasteries, nunneries, and boarding schools is often listed as a determinant of TB in Tibetan communities.^26,4^ The close proximity in which people reside, eat, and pray can accelerate the spread of TB. What is missing, however, are the core reasons for this problem. Many Tibetans turn to sending their children to monasteries and boarding schools as a form of economic relief. Parents often send their children to monasteries where they are fed, educated, and housed free of cost. They also learn more about Tibetan culture and religious practices. Boarding schools become a space for parents to send their children while they are engaging in seasonal work such as sweater selling or farming. How would life look different if parents were able to send their children for day school, where the risk for TB is much lower, compared to boarding schools? How may a PAR approach to this issue uncover such nuances?

The isolated (physical and social) nature of Tibetan settlements in India is commonly cited as a determinant of TB.^3,4^ What is often missing, however, is the early historical context of the creation of these very settlements. When thousands of Tibetans first fled into India, the Indian government created two transit camps.^4^ These camps were originally designed to house a small number of people. Over time, however, these camps became overcrowded, leading to the rapid spread of diseases such as dysentery and TB.^4^ We must not brush aside the intergenerational impacts of the creation of such camps on the Tibetan community. As Maani and colleagues^27^ state, to improve global health equity, we need a more contextual understanding of between- and within-country inequalities, with a particular focus on cultural and historical backgrounds and experiences.

I feel that these nuances are missing from the TB discourse in the Tibetan refugee community. My positionality and lived experience as a Tibetan scholar have privileged me with the ability to find gaps in research that non-Tibetan scholars simply may neither seek nor understand. A more thorough, person-centered and contextual understanding of the TB experience of Tibetan refugees is critical to eradicating TB in this pocketed community and other similar communities globally.

## Reflexivity and Positionality as a Decolonial Tool

Reflexivity is a critical practice and characteristic for global health researchers, closely tied to positionality. However, positionality is also dynamic, and it evolves over time and across different contexts. Positionality is made up of various, intersecting identities that inform the way we engage with and understand the world around us. It can thus act as a “confession of privilege” to reveal the unequal power dynamics that exist in the research setting between the researcher and the researched subject.^28^ Positionality also makes clear the subjectivity that may arise from one's positionality. For example, the research design and methods are connected to one's worldview and are therefore inadvertently influenced by the positionality of a researcher.^29^ Practicing and exercising reflexivity is a critical and intentional decolonial tool as it fosters a level of self-awareness that may not otherwise arise during the research process.

Saleh and colleagues^30^ argue for the standardization of the submission of reflexivity/positionality statements by authors alongside manuscripts. The authors believe that, similar to a declaration of competing interest, this practice will encourage researchers to consider the equity of partnerships. It also inadvertently forces journals to consider holistically the importance of equitable partnerships collaborative projects.

My positionality as a Tibetan scholar born in Nepal but based in the Global North provides me with access to resources, networks, and funding opportunities that may not necessarily be available for Tibetan scholars in India. I believe that it is my responsibility to involve the local Tibetan community in my research agenda. My insider–outsider positionality as a Tibetan person does not automatically negate any need to involve the community in my work. Although I am a part of the global Tibetan diaspora, Tibetan scholars in India may better understand the realities faced by Tibetans living in India. Their proximity, both physical and virtual, to the community and engagement with issues provide them with deep insight into the daily lives of Tibetans in India. My perspectives are indeed valuable but are instead shaped by my engagement with the Tibetan community in Canada, which is much different. My academic experience as a scholar based in the Global North does not inherently make me better positioned to conduct research on Tibetan issues in India compared to a Tibetan scholar from an institution in India. While it is also true that Tibetan scholars in India may not inherently be better positioned to research topics on Tibetan peoples, the disparity in opportunity complicates their chances of accessing higher education and research.

While scholars from the Global South may face financial and logistical challenges when publishing, they also may face forms of epistemic injustices rooted in their positionality as scholars from the Global South.^31^ This injustice may manifest in the discrediting of these scholars as experts or knowledge holders and producers and the complete erasure from authorship despite major contributions. It can also result in the dismissal of credibility and expertise by editors and reviewers of academic journals, a term designated as “editorial racism”.^32^ That is, publications led solely by authors from the Global South are not credible and are made more “credible” in collaboration with scholars from the Global North.^33^ The underlying assumption here is that the knowledge created by and within the Global South does not meet the standards of the Global North—that the pursuit of academics in the Global South should adhere to the standards of the Global North.^34^ This is a deeply colonial and racist mindset that has entrenched academia, or a coloniality of knowledge that centers Eurocentric epistemology.

## Framing the Issue: Beyond Academia

By no means am I arguing for the exclusion of non-Tibetan scholars from studies done on the Tibetan community. As Abimbola states,^17^ “The foreign gaze is inevitable. In a globalizing world, our destinies are interlinked, and the origins of and solutions to delivery problems in global health can be local or foreign.” I instead argue for equal access to opportunity for Tibetan scholars, both in the Indian contexts and beyond. Abouzeid and colleagues^35^ list three forms of barriers that undermine health research disparities in the global south, including researcher-level barriers (lack of mentorship, poor funding for students), institutional barriers (under resourced facilities, weak research infrastructures and collaboration opportunities), and structural barriers (lack of political will, political instability). While there are a plethora of reasons explaining the disparity in scholarship from the Global South, I suggest we dig deeper into structural barriers that hinder academic growth for Global South scholars. For the Tibetan diaspora, the factors are vast and diverse.

I argue that the exclusion of Tibetan scholars from India from mainstream academia can be attributed to the limitations on the social, economic, and political growth of Tibetans in India. The lack of opportunity for Tibetan scholars globally parallels the lack of opportunity for Tibetan scholars in India. The problem of the marginalization of scholars from the Global South, however, cannot be separated from its social, financial, and economical contexts. In the case of Tibetan refugees in India, we must focus on addressing the precarious nature of one's legal status and its implications for educational pursuits.

As India does not have a refugee framework or law, most Tibetans in India are not citizens; rather, they are given the legal designation of “foreigner,” or they simply exist without legal documents.^36^ The “foreigner” status puts limitations on one's ability to grow within fields such as academia.^5^ Most Tibetans in India engage in precarious forms of employment and higher education is rarely sought.^6^ Thus, Tibetan “refugees” are not granted the same opportunities as are Indian citizens. For example, postsecondary education is a difficult pursuit for many Tibetans due to various reasons, including costs (more expensive due to “foreigner” status in India), lack of available seats for “foreigners,” insufficient scholarships to pursue higher education, and more.^37^ A 2009 demographic survey conducted by the Tibetan government-in-exile found that only 13 percent of the literate Tibetan population had university or college degrees.^37^ Perhaps the lack of Tibetan academics studying TB and other issues that disproportionately burden Tibetan people stems from challenges that extend beyond academia and the borders of global health.

The issue of the marginalization of scholars from the Global South cannot be addressed by “fixing” academia; it ultimately begins with addressing systemic *global* inequities. The issue of health inequities, analogously, cannot be addressed by “fixing” health *care*. It begins with addressing the unique social determinants of health that are context dependent. The various intersecting identities of Tibetans as either refugees, foreigners, or existing without documents, informed by other social locations such as gender, age, education status, and income, among others, may restrict their opportunity to pursue higher education and employment.

I must emphasize here that Tibetan refugees in India are immensely grateful to the Indian state for their continued acceptance of the Tibetan peoples. However, acknowledging such tensions is imperative. The issue of the lack of Tibetan scholars engaging in research about the Tibetan community, then, is one that extends beyond academia. On a global scale, the continued devaluation of scholars from the Global South adds to the marginalization of this group.

However, rather than focusing on the perceived weaknesses of Tibetan refugees in India, a strengths-based approach is key to fostering leadership and growth within this community. The recognition and acknowledgement of the capabilities of Tibetan scholars must center this approach. The devaluing and deemphasizing of research led by scholars from the Global South is simply unethical. It represents a colonial mindset that has been embedded into academia since its inception. It is predicated on the unacceptability of non-Western knowledge and methods of knowledge production.

Scholars from the Global North who wish to conduct research on the Tibetan refugee community, for example, should incorporate Tibetan voices into all parts of the research process, from start to finish. Community partnerships and acknowledgements should be at the forefront of any research process. But without structures and institutions to hold scholars accountable, can this change come to fruition? Must scholars from the Global South always rely on cross-border collaborations for their work to be valued? Must scholars from the Global South attend and work for institutions in the Global North for their voices to be heard and be considered legitimate? The marginalization of scholars from the Global South must be framed within the larger-scale issues of global social inequities.

## Recommendations

The diverse legal status of Tibetan refugees in India, ranging from citizen to foreigner to those with no formal documentation, along with the precarious position of the CTA in India, complicates any recommendations one may give. However, I will position my recommendations toward the CTA and the Tibetan diaspora. I believe that the scholars and institutions in the Global North can also benefit from these recommendations. There is a body of knowledge about various steps scholars can take to decolonize Global Health. These recommendations are directed toward the Tibetan diaspora, and it is a conscious choice. These recommendations are grounded in my belief that the most effective support for Tibetan scholars comes from recognizing and building upon their unique strengths and cultural insights.

1. Community engagement: Mandate and assess the involvement of the Tibetan community in research projects conducted on Tibetans. This may include a more rigorous ethics process for scholars from the Global North wishing to conduct research on the Tibetan community. A standardized ethics process with a section on the steps taken to involve the Tibetan community must be included.
2. Invest in Tibetan academics to pursue higher education: This must be a concerted effort from both the CTA and the GoI and includes scholarship programs, more seats for Tibetan students in Indian institutions, funds for publishing and attending conferences, and funds for other academic activities.
3. Collaboration between Tibetan institutions and Indian universities: Foster partnership between Tibetan institutions and Indian education institutes for joint programs, research projects, and faculty exchanges.
4. Opportunities for knowledge exchange for academics: This may include organizing regular academic conferences and workshops to promote networking among Tibetan and non-Tibetan scholars, specifically held in India and Nepal.
5. Digital dissemination hubs: The CTA may choose to develop an online platform for sharing research projects and publications lead by or authored by Tibetan scholars in the global diaspora.
6. Mentorship programs: Establish programs whereby emerging Tibetan scholars or those who show interest in higher education are paired with Tibetan scholars from the global Tibetan diaspora.
